# Multiclass CNN Approach for Automatic Classification of Dolphin Vocalizations

**DOI:** 10.3390/s25082499

**Published:** 2025-04-16

**Authors:** Francesco Di Nardo, Rocco De Marco, Daniel Li Veli, Laura Screpanti, Benedetta Castagna, Alessandro Lucchetti, David Scaradozzi

**Affiliations:** 1 Dipartimento di Ingegneria dell’informazione, Università Politecnica delle Marche, 60131 Ancona, Italy; l.screpanti@staff.univpm.it (L.S.); b.castagna@staff.univpm.it (B.C.); 2 Institute of Biological Resources and Marine Biotechnology (IRBIM), National Research Council (CNR), 60125 Ancona, Italy; rocco.demarco@cnr.it (R.D.M.); daniel.liveli@cnr.it (D.L.V.); alessandro.lucchetti@cnr.it (A.L.); 3 ANcybernetics, Università Politecnica delle Marche, 60131 Ancona, Italy; 4 National Biodiversity Future Center, 90133 Palermo, Italy

**Keywords:** convolutional neural networks, deep learning, dolphins, passive acoustic monitoring

## Abstract

**Highlights:**

**What are the main findings?**

- A CNN was developed for multiclass classification of dolphin vocalizations.
- The model achieved a 95.2% mean accuracy with a mean F1-score of 87.8%.

**What is the implication of the main finding?**

- This approach enhances the passive acoustic monitoring of dolphin vocalizations.
- It provides a scalable AI-based solution for marine bioacoustics research.

**Abstract:**

Monitoring dolphins in the open sea is essential for understanding their behavior and the impact of human activities on the marine ecosystems. Passive Acoustic Monitoring (PAM) is a non-invasive technique for tracking dolphins, providing continuous data. This study presents a novel approach for classifying dolphin vocalizations from a PAM acoustic recording using a convolutional neural network (CNN). Four types of common bottlenose dolphin (*Tursiops truncatus*) vocalizations were identified from underwater recordings: whistles, echolocation clicks, burst pulse sounds, and feeding buzzes. To enhance classification performances, edge-detection filters were applied to spectrograms, with the aim of removing unwanted noise components. A dataset of nearly 10,000 spectrograms was used to train and test the CNN through a 10-fold cross-validation procedure. The results showed that the CNN achieved an average accuracy of 95.2% and an F1-score of 87.8%. The class-specific results showed a high accuracy for whistles (97.9%), followed by echolocation clicks (94.5%), feeding buzzes (94.0%), and burst pulse sounds (92.3%). The highest F1-score was obtained for whistles, exceeding 95%, while the other three vocalization typologies maintained an F1-score above 80%. This method provides a promising step toward improving the passive acoustic monitoring of dolphins, contributing to both species conservation and the mitigation of conflicts with fisheries.

## 1. Introduction

Monitoring dolphins in the open sea is crucial for understanding their behavior, population dynamics, and overall health, as well as for assessing the impact of human activities on their habitats [1,2]. One of the most effective techniques for this purpose is Passive Acoustic Monitoring (PAM) [3,4]. This non-invasive approach uses underwater microphones (hydrophones) with suitable recording systems to detect and capture dolphin vocalizations, allowing researchers to track their presence and movements without direct interference [5]. PAM is particularly valuable in remote or deep-sea areas as well as in unfavorable weather conditions where visual observation is challenging. By providing continuous and reliable data, this technique plays a vital role in conservation efforts, helping to mitigate threats such as noise pollution and habitat degradation.

Common bottlenose dolphins are known to adopt a wide range of vocalizations depending on the context in which they are used [6]. Whistles are narrowband, omnidirectional acoustic signals with frequency modulation, serving as an essential communication tool. They convey information about the environment, social interactions, and individual identity [7]. Echolocation clicks and burst pulse sounds are broadband impulsive vocalizations (mostly ultrasonic) that differ in the duration of inter-click intervals, which are shorter for burst pulse sounds. Dolphins typically emit these vocalizations for echolocation purposes during navigation and hunting [8]. Feeding buzzes are also impulsive vocalizations but are characterized by lower-frequency content than echolocation clicks and burst pulse sounds [9].

Conventional acoustic approaches for identifying dolphin vocalizations are typically based on an algorithmic analysis of audio spectrograms [10,11,12]. The audio spectrogram visually represents sound, showing how its frequency content varies over time. Recent studies, including our own, have tried to employ the potential of machine/deep learning algorithms to enhance the efficiency of monitoring and detecting marine mammal presence by identifying their vocalizations [13,14,15,16,17,18,19,20]. Some of these analyses focused on the identification of whistles alone, disregarding impulsive vocalizations [16,17,18,19,20]. Others were more oriented towards species identification [13,14,15]. Most of these studies employed convolutional neural networks (CNNs), highlighting their suitability as tools for this type of classification [13,14,15,16,17,19]. Specifically, CNNs were employed to classify whale vocalizations, significantly reducing the false-positive rate compared to traditional algorithms [17,21]. Moreover, it was reported that CNNs outperformed four traditional methods for detecting dolphin echolocation clicks [22]. Ultimately, the outcomes of Nur Korkmaz et al. suggested the superiority of CNNs over human experts in dolphin call detection accuracy [16].

However, to the best of our knowledge, no approach in the literature has definitively addressed the complex challenge of classifying the wide range of dolphin vocalizations. Challenges such as the diversity of species and their vocalizations, technological limitations, high levels of ambient noise, and limited availability of data continue to pose significant obstacles to achieving reliable results and a high performance in neural network models. Moreover, dolphins swimming freely in the open sea can produce different vocalizations depending on the circumstances. Typically, common sounds like whistles and echolocation clicks are often overrepresented, leading to an inherent imbalance in most datasets of dolphin vocalizations, where less frequent vocalizations, such as feeding buzzes, appear in much smaller numbers. Given that convolutional neural networks perform best when trained on large and balanced datasets [23], this imbalance poses an additional challenge that could negatively impact the classification process. Therefore, additional quantitative studies on the topic are recommended.

Thus, the present study was designed to introduce a novel approach based on CNNs for the multiclass classification of four distinct vocalizations of common bottlenose dolphin (*Tursiops truncatus*) from underwater audio recordings: whistles, echolocation clicks, burst pulse sounds, and feeding buzzes. These vocalizations were also distinguished from background noise. An analysis of vocalization spectrograms revealed that dolphin whistles appear as distinct, continuous lines developing horizontally. Differently, impulsive vocalizations develop vertically, such as echolocation clicks and burst pulse sounds. The present work aims to leverage these peculiar differences among the signal frequency content to enhance the recognizability of each specific vocalization, filtering out undesired signals from the spectrograms to be fed into the CNN during the training phase. Specifically, vertical edge-detection filters were used to remove from the spectrograms the disturbing vertical components within vocalizations characterized by a horizontal waveform (whistles) [24,25]. Similarly, horizontal edge-detection filters were applied to cancel the disturbing horizontal components within the vocalizations that develop vertically (impulsive vocalizations). Our intention is that this approach will improve the training of the neural network and, consequently, the identification performance.

## 2. Materials and Methods

### 2.1. Signal Collection

Acoustic signals were acquired on 20–21 November 2021, at the Oltremare thematic marine park in Riccione, Italy, where seven bottlenose dolphins (*Tursiops truncatus*) were free to swim between different interconnected pools. The animals’ care was by institutional guidelines. Recording sessions were performed using a UREC 384K autonomous underwater recorder (Dodotronic and Nauta), equipped with an SQ26-05 hydrophone (Sensor Technology, Collingwood, ON, Canada). The hydrophone was reported to have a sensitivity of −193.5 dB re 1 V/μPa @ 20 °C at 1 m between a few Hz and 28 kHz. The sensitivity profile applicable to the entire SQ26 series is available at the link reported in [26]. Moreover, the literature provides further indication regarding the effective response of the hydrophone at higher frequencies. Palmero et al. reported a hydrophone sensitivity of −169 dB re 1 V/μPa at 1 m up to 50 kHz [27]. Moreover, the sensitivity of the hydrophone was reported to be −169 ± 5 dB re 1 V/µPa at 1 m between 10 Hz and 96 kHz [28]. The signals were acquired at a sampling frequency of 192 kHz and stored as 16-bit wave files with a duration of 5 min each. The recording was carried out in passive mode without any interference with the wellbeing of the animals. As an additional precaution, during the placement and retrieval of the recorder, the dolphins were moved to other tanks.

All recordings were analyzed by an expert PAM operator, focusing on identifying and labeling whistles, click trains, burst pulse sounds, feeding buzzes, and their starting and ending events by visual inspection of the audio spectrogram of the signals. The release of the dataset was in preparation; however, the data are already available by contacting the authors of this manuscript.

### 2.2. Data Preparation

Before feeding the data into the classifier, each recording underwent min–max normalization, mapping the values to the [0–1] range. To train the CNN, each 5 min acoustic recording (wave file) was split into segments of 0.8 s duration, centered on the vocalization identified and labeled by a PAM expert. If the labeled signal lasted more than 0.8 s, it was divided into 0.8 s segments, with a 50% overlap, until the entire signal was covered. Thus, all input signals used to train the classifier had a uniform length of 0.8 s. For each of these 0.8 s segments, a gray-scale spectrogram was generated with dimensions of 300 × 150 pixels. The gray-scale spectrogram was band-pass filtered between 3 kHz and 96 kHz. The gray-scale spectrograms were subsequently labeled by the PAM operator with specific tags indicating the presence of whistles (class 1), echolocation clicks (class 2), burst pulse sounds (class 3), and feeding buzzes (class 4). The spectrograms in which none of the aforementioned vocalizations were identified were considered as noise and labeled with the tag “class 0”. The labeling was performed by an expert PAM operator using the open-source software Audacity version 3.2.4 [29]. This procedure aimed to identify the Ground Truth, which is the set of real data essential for training and validating the models.

Then, a Sobel filter (kernel size = 7) was implemented to perform edge detection on the spectrograms [30] to improve the CNN classification performance. The Sobel filter was applied in the vertical direction for whistle vocalizations and for noise in order to remove the disturbing vertical components. It was used in the horizontal direction for spectrograms depicting echolocation clicks, burst pulse sounds, and feeding buzzes to cancel the disturbing horizontal components. The filter selection was performed manually. To evaluate the sensitivity of the CNN performance to changes in kernel size, preliminary experiments were performed, considering five different kernel sizes. A simplified network was trained (with two convolutional layers, reduced filter counts of 16 and 32, and a dense layer reduced to 64 neurons) for the recognition of whistles only. The dataset, composed of 4000 positive images (whistles) and 4000 negative images (ambient noise and other vocalizations), was split into three portions: 65% for training, 10% for validation, and the remaining 25% for testing. Spectrogram images were generated and used after applying the Sobel filter with kernel sizes of 3 × 3, 5 × 5, 7 × 7, 9 × 9, and 11 × 11. The results are presented in Table 1.

The results in Table 1 indicate a high performance across all tests despite the significant simplification of the model. Moreover, these outcomes highlight that the network performance remains virtually unchanged with variations in kernel size; this is particularly true for crucial parameters such as accuracy and F1-score. Thus, in the present study, a 7 × 7 kernel was employed, following the procedure described in [19].

The multiclass CNN model was trained using the filtered spectrograms of the Oltremare dataset. This dataset of spectrograms was randomly split into 10 equal folds. Following the so-called 10-fold cross-validation procedure, labeled spectrograms from 9 out of 10 folds were used to train the model [31]. The labeled spectrograms from the remaining single fold were used as a test set. The spectrograms used during the test had never been used to train a CNN. The procedure was performed ten times, each time using a different fold as a test set. In this way, ten different models were trained. Classification performances were provided as the mean value (±standard deviation, SD) over the ten folds.

### 2.3. The Convolutional Neural Network

A Convolutional Neural Network is a class of deep feed-forward artificial neural networks primarily used for image analysis. These models have been widely adopted for speech recognition and audio-related studies [32]. The CNN processes 300 × 150-pixel gray-scale spectrograms in input, using a sequential structure with convolutional and pooling layers followed by dense layers for classification. The CNN is composed of three convolutional layers of 32, 64, and 128 filters, respectively (6 × 3 kernel, rectified linear units, ReLU, activation). Each layer is followed by a max pooling layer with a 2 × 2 window to reduce the spatial dimensionality of the output, decreasing computational complexity and helping to prevent overfitting. The output from the convolutional and pooling layers is flattened into a one-dimensional vector that is processed by a dense layer with 128 units and ReLU activation. The final dense layer has a single unit with a Softmax activation function configured for multiclass classification. The output will be a probability distribution indicating the likelihood of belonging to one of the five classes: noise, whistles, echolocation clicks, burst pulse sounds, or feeding buzzes.

## 3. Results

**Figure 1:**
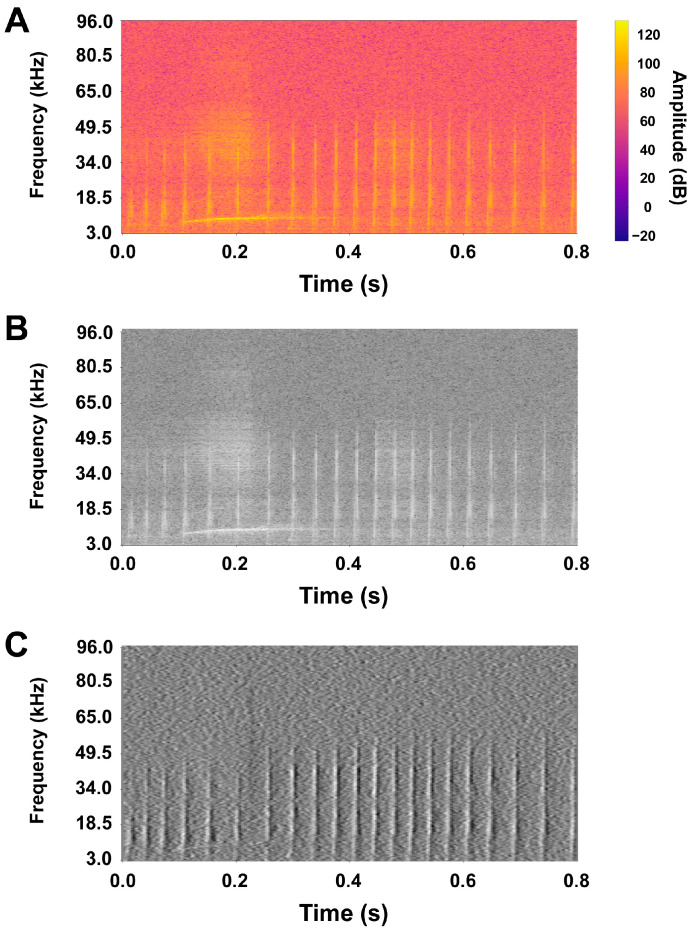
(**A**) depicts the color spectrogram of an exemplifying click train. (**B**) reports the same spectrogram in grayscale. (**C**) shows the spectrogram after horizontal Sobel filtering.

In total, the dataset used contained 3000 spectrograms representing whistles (class 1), 2000 representing clicks (class 2), 1400 representing burst pulse sounds (class 3), 420 for feeding buzzes (class 4), and finally, 3000 for noise (class 0). Figure 1 shows an example of a spectrogram of a dolphin whistle before (panels A and B) and after (panel C) the application of the horizontal Sobel filter.

**Figure 2:**
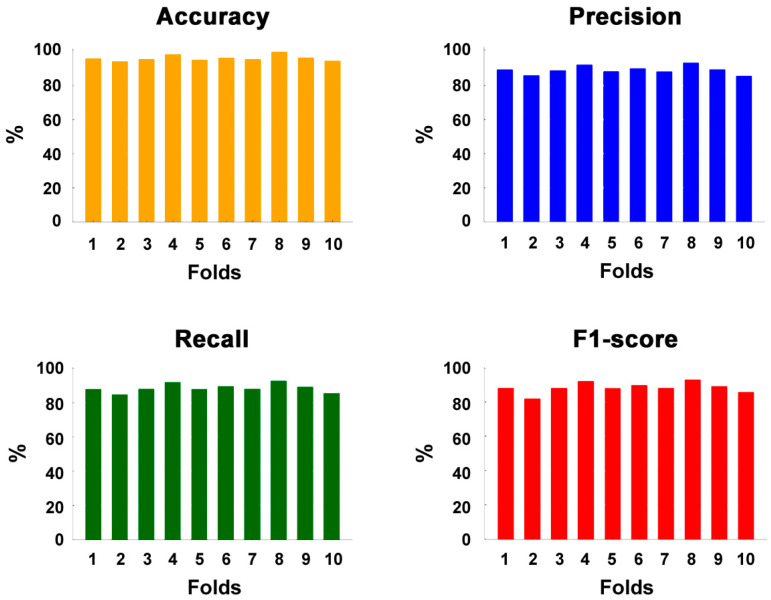
Percentage performance metrics of the CNN model across 10 folds, showing accuracy in orange, precision in blue, recall in green, and F1-score in red. The percentages were computed on the entirety of the spectrograms, regardless of the vocalization represented.

A comparison between panels B and C shows how the application of the horizontal Sobel filter allows for the removal from the spectrogram of disturbing horizontal components, highlighting the vocalizations that develop vertically, such as echolocation clicks and burst pulse sounds. The low-frequency horizontal component centered around 0.2 s, which is evident in the image shown in panels A and B, almost disappears in the filtered image presented in panel C. The overall classification performances in terms of accuracy, precision, recall, and F1-score are reported in Figure 2 for all ten folds.

Figure 2 shows that, for all performance parameters, there is limited variability across the folds, with values ranging between 92.9% and 97.1% for accuracy, between 84.2% and 93.2% for precision, between 83.6% and 92.8% for recall, and between 81.8% and 92.8% for the F1-score. The mean (±SD) values over ten folds of accuracy, precision, recall, and F1-score in each class are reported in Table 2. The average values across all classes are shown in the last row of the table, showing that the mean accuracy was higher than 95%, and the mean precision, recall, and F1-score were close to 90%.

**Figure 3:**
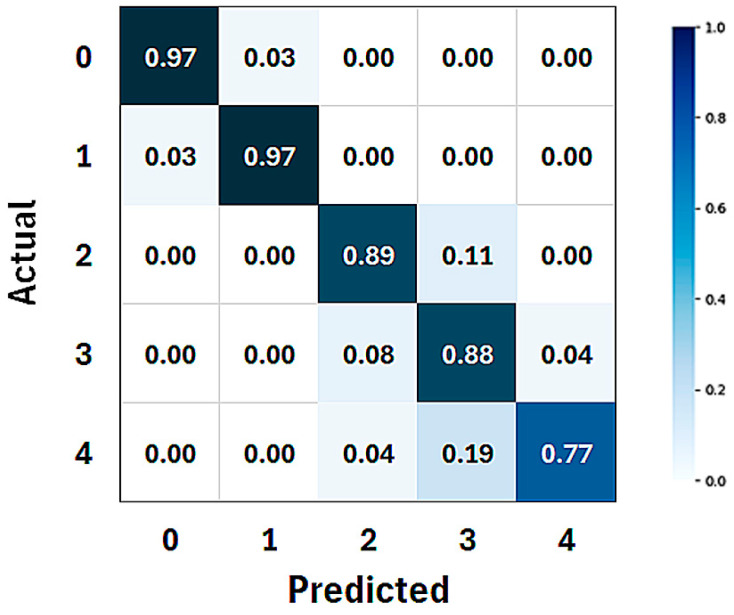
Confusion matrix. Data are reported as mean values over ten folds and normalized between 0 and 1.

The confusion matrix highlights how predictions are distributed across the five classes. The confusion matrix with color-scale-normalized average values over 10 folds is reported in Figure 3. True positive values for the five classes are reported in the diagonal of the matrix.

On average, 97% of all whistles were correctly detected (matrix element (1,1)). Echolocation clicks and burst pulse sounds were identified in 89% (matrix element (2,2)) and 88% (matrix element (3,3)) of the occurrences, respectively. Only for feeding buzzes, this percentage dropped below 80% (matrix element (4,4)).

## 4. Discussion

Deep neural networks have demonstrated great potential in underwater acoustic monitoring, particularly CNNs [13,14,15,16,17,19,21]. The present study introduces a new deep-learning-based approach to identify the different vocalizations of common bottlenose dolphins from underwater audio recordings, providing more comprehensive acoustic monitoring of dolphin behavior. Dolphin vocalizations are complex and highly variable [33]. Therefore, they are typically analyzed in the frequency domain using spectrograms [10,11,12]. The idea of the current work was to classify the different vocalizations based on the waveforms present in the spectrograms. Specifically, vocalizations have been classified into five different categories: noise (or absence of vocalization), whistle, echolocation click, burst pulse sound, and feeding buzz [7,8,9].

The idea behind the current study is to enhance the recognizability of dolphin vocalization waveforms in spectrograms by applying the Sobel filter. This filtering procedure is characterized by the ability to highlight edges and contours in an image [24,25]. Echolocation clicks, burst pulse sounds, and feeding buzzes are broadband impulsive vocalizations (mainly ultrasonic) that develop vertically in the 96 kHz spectrogram [8]. Thus, using a horizontal Sobel filter, the horizontal components of the audio signal could be deleted from the spectrogram, thereby highlighting only the vertical components. An example of this is reported in Figure 1, which highlights the comparison between an unfiltered click train (panel B) vs. a Sobel-filtered one (panel C). In a spectrogram, dolphin whistles primarily develop horizontally [7]. By applying the vertical Sobel filter, it is possible to remove the vertical components of the audio signal in the spectrogram, thereby isolating the horizontal components of the image, which correspond to the typical frequency modulation patterns of the dolphin whistles. To the best of our knowledge, this was the first attempt to use a Sobel filter to enhance the recognizability of dolphin vocalizations in a multiclass neural network.

The effectiveness of this approach was supported by the classification performance reported for each fold in Figure 2 and on average in the last row of Table 2, suggesting that the presented results may serve as an encouraging starting point for the multiclass classification of dolphin vocalizations. On average, the accuracy exceeded the remarkable threshold of 95%. The other three performance metrics were very close to 90%, which is a very good benchmark for multiclass classification problems. Moreover, the low values of the standard deviations indicated that the model performed consistently across different data subsets (Table 2). This suggests that the observed performance accurately reflected the model capabilities rather than being influenced by random variations in the data, demonstrating the high consistency and reliability of the model performance. These values were not far from those reported in the few studies on dolphin vocalization classification. Jin et al. achieved a mean accuracy of 89%, precision of 96%, recall lower than 80%, and F1-score of 86% with their CNN-based semantic segmentation model for whistle profile extraction [18]. Nur Korkmaz et al. tested two architectures: vanilla CNN and a pre-trained CNN based on VGG16 architecture [16]. The best-performing model accomplished an average identification accuracy of 92.3%, precision of 90.5%, and recall of 89.6% (the F1-score was not reported, but presumably it was around 90%). It is worth noting, however, that these studies report results for the binary classification of whistles, which is a significantly simpler task compared to the multiclass classification presented here. We also attempted to identify the presence of whistles alone in a recent paper [19]. The results of this binary classification yielded an accuracy of 87.0%, precision of 91.7%, recall of 81.3%, and F1-score of 86.2%. The improved results obtained in this study for whistle identification (Class 1) were due to the use of pre-processing with the Sobel filter, which was not applied in the previous study.

The results stratified by class in the confusion matrix in Figure 3 and in Table 2 also seem to confirm the effectiveness of this approach. On average, nearly all whistles (97%, matrix element (1,1) in Figure 3) were correctly identified by the CNN. This result, together with an accuracy of 97.9% and an F1-score of 95.0% reported for this class in Table 2, suggests that the distinct spectral patterns of whistles make them easier for the CNN to identify. The other three vocalizations are classified with a lower performance compared to whistles. This is likely because all three signals appeared on the spectrogram as a series of consecutive pulses. Therefore, despite these pulse trains differing in the inter-click interval and frequency band, the CNN may sometimes fail to correctly identify the vocalization due to the similarity between the impulsive waveforms. Specifically, echolocation clicks appeared to be the best-identified impulsive vocalizations, as reported in the third row of Table 2 and the (3,3) element of the matrix shown in Figure 3. The estimated performance for classes 3 (burst pulse sounds) and 4 (feeding buzzes) showed a very similar pattern, with accuracy and F1-score values being highly comparable (Table 2). However, the confusion matrix seemed to suggest that the CNN struggled only with identifying feeding buzzes, although the percentage was still slightly below 80%. The main reason for this was likely the limited number of spectrograms used in both the training and test phases compared to the other vocalizations [23]. Unfortunately, this is an intrinsic limitation of the dataset used, which contained a relatively small number of feeding buzzes since these vocalizations are emitted less frequently by dolphins and only during feeding phases. However, this is consistent with most datasets on dolphin vocalizations, which, for the reasons mentioned above, tend to be biased towards more common vocalizations such as whistles and echolocation clicks, to the detriment of more specific and less frequent ones like feeding buzzes. As a result, working with imbalanced datasets has become standard practice in this field. Another possible reason is their similarity to other vocalizations, as their waveforms closely resemble those of clicks and burst pulse sounds.

However, it is important to acknowledge that the dataset has inherent limitations that may contribute to lower classification performance (particularly recall) for feeding buzzes. Specifically, the data were collected in a controlled dolphin pool environment where dolphins were trained to eat in response to task completion or at specific feeding times. This structured feeding regimen differs from the more variable and spontaneous feeding behaviors observed in the wild, leading to a lower number of recorded feeding buzzes compared to other vocalizations. Therefore, testing the CNN on a dataset collected in a natural marine environment could help address the dataset imbalance. This possibility will be considered in future studies by this research group.

Moreover, from the analysis of the confusion matrix (Figure 3), it emerges that the reduced classification performance for feeding buzzes (class 4) was mainly due to the fact that a CNN misinterprets some feeding buzzes as burst pulse sounds, because these vocalizations share some characteristics, such as rapid pulse repetition rates and similar energy distributions across frequencies. These similarities can lead to confusion, as the classifier may struggle to differentiate between them based on the extracted features. In this study, we did not apply data augmentation or oversampling techniques to balance the dataset in order to improve feeding-buzz-classification performances. Our goal was to first assess the classifier performance on real, unaltered data before considering artificial expansion methods. However, we admit that class imbalance may lead to biased model predictions, favoring the majority class while reducing sensitivity to the minority class.

Some possible approaches that take into account the specific characteristics of the different types of signals can be attempted to address this problem. A promising approach could be to implement a preprocessing step that separates feeding buzzes based on frequency content. Feeding buzzes typically exhibit lower dominant frequencies (<30 kHz) than echolocation clicks and burst pulse sounds (>40 kHz). By applying frequency-based filtering techniques or spectral subtraction, feeding buzzes could likely be isolated from these other signals before classification, potentially improving recall. Another approach, which can also be integrated with the previous one, could be based on the observation that feeding buzzes exhibit different values of the inter-click interval (ICI), which is defined as the absolute time distance between two consecutive peaks. Specifically, in the feeding buzzes, the ICI is reported to be <10 ms, while the other two impulsive vocalizations are characterized by significantly larger ICIs, even by an order of magnitude [34,35,36]. These possible solutions will be investigated in our future studies.

On the other hand, the model was able to correctly classify almost all spectrograms that contained no vocalizations, which we referred to as noise (matrix element (0,0) in Figure 3). This, together with the high performances reported in the first row of Table 2, is a highly significant result when considering the use of this approach to detect the presence of dolphins based on any of their vocalizations. An amount of 97% of spectrograms correctly identified as noise ensures that the model rarely misinterprets ambient noise as a dolphin presence. It is true that this outcome was achieved in a dataset recorded in a highly controlled environment, such as a dolphin pool, and that performance would likely degrade in open sea conditions. However, it remains a very encouraging starting point.

The dataset used in this study was collected in a controlled pool environment. In natural habitats, CNN-based classification performance would likely be lower. Several factors could contribute to this expected decrease in performance. First, open-sea environments introduce highly variable and unpredictable background noise, including biotic sources (e.g., vocalizations of other marine species) and abiotic sources (e.g., vessel traffic, wave action, and turbulence). This additional noise could interfere with the extraction of relevant features from the spectrograms, making it harder for the model to distinguish between different bio-acoustic signals [37]. Moreover, variability in dolphin behavior, such as differences in vocalization patterns depending on context, could further contribute to classification challenges. Nevertheless, the correct application of the vertical and/or Sobel filters could help reduce environmental noise, thereby minimizing its impact on the model performance. A reduction in performance is certainly expected, but not too significantly, thanks to the filtering applied to the spectrograms. Applying a CNN with a Sobel filter to data recorded in natural environments is a promising direction to be explored in future work. However, for the moment, it was considered valuable to start with controlled data to establish a baseline reference for CNN performance before extending the approach to more complex and variable real-world conditions.

These promising results were likely achieved thanks to the consistent procedure used to generate spectrograms for both the test and training sets, centering the vocalizations within the 0.8 s window. Although the spectrograms used during the testing phase had never been used to train a CNN, both the training and test spectrograms were processed to center the vocalization in the image. This procedure increased dataset consistency and improved network performance. However, this approach is only feasible in scenarios where post-processing the recorded signal is possible. Thus, further studies are going to be focused on testing an alternative image generation method that attempts to simulate real detection conditions, allowing the convolutional neural network to be evaluated in dynamic conditions, simulating the real-time identification and classification of dolphin vocalizations. Future work will also explore comparisons with other deep learning architectures to further validate the present findings. However, for the moment, we believe that the present study presents a useful analysis to effectively support the potential benefits of integrating Sobel filtering into the spectrogram-based classification pipeline.

## 5. Conclusions

Overall, this study represents a step forward in the use of artificial intelligence for marine mammal monitoring, offering a foundation for further advancements in the automated analysis of dolphin vocalizations. The application of edge-detection filters to spectrograms seems to be an effective preprocessing technique, enhancing the model ability to distinguish among different vocalization types while reducing the impact of unwanted noise. Future work should focus on addressing class imbalances through data augmentation techniques or by collecting additional recordings from natural marine environments. Additionally, testing the model in real-time applications under varying acoustic conditions will be essential to assess its robustness in field deployments.

## Figures and Tables

**Table 1:** CNN performances in function of kernel size of the Sobel filter.

Kernel Size	Accuracy	Precision	Recall	F1 Score
3 × 3	97.3%	98.4%	96.3%	97.3%
5 × 5	97.5%	98.2%	96.8%	97.5%
7 × 7	97.4%	97.8%	97.0%	97.4%
9 × 9	97.4%	97.5%	97.3%	97.4%
11 × 11	97.2%	97.5%	96.8%	97.2%

**Table 2:** Mean classification performances.

Class	Accuracy (%)	Precision (%)	Recall (%)	F1-Score (%)
0	97.9	95.0	95.6	95.0
1	97.9	95.2	95.0	95.0
2	94.5	85.9	87.6	86.6
3	92.3	77.0	88.1	81.9
4	94.0	92.6	76.2	83.0
Mean	95.0 ± 1.1	89.1 ± 2.8	89.0 ± 2.7	88.0 ± 3.1

## Data Availability

The release of the dataset is in preparation; however, the data are already available by contacting the authors of this article.

## Abbreviations

The following abbreviations are used in this manuscript:

CNN	Convolutional Neural Network
PAM	Passive Acoustic Monitoring
SD	Standard Deviation
